# Latent space improved masked reconstruction model for human skeleton-based action recognition

**DOI:** 10.3389/fnbot.2025.1482281

**Published:** 2025-02-12

**Authors:** Enqing Chen, Xueting Wang, Xin Guo, Ying Zhu, Dong Li

**Affiliations:** ^1^School of Electrical and Information Engineering, Zhengzhou University, Zhengzhou, China; ^2^State Grid Henan Electric Power Company Information and Communication Branch, Zhengzhou, China

**Keywords:** human skeleton-based action recognition, variational autoencoder, vector quantized variational autoencoder, masked reconstruction model, self-supervised learning

## Abstract

Human skeleton-based action recognition is an important task in the field of computer vision. In recent years, masked autoencoder (MAE) has been used in various fields due to its powerful self-supervised learning ability and has achieved good results in masked data reconstruction tasks. However, in visual classification tasks such as action recognition, the limited ability of the encoder to learn features in the autoencoder structure results in poor classification performance. We propose to enhance the encoder's feature extraction ability in classification tasks by leveraging the latent space of variational autoencoder (VAE) and further replace it with the latent space of vector quantized variational autoencoder (VQVAE). The constructed models are called SkeletonMVAE and SkeletonMVQVAE, respectively. In SkeletonMVAE, we constrain the latent variables to represent features in the form of distributions. In SkeletonMVQVAE, we discretize the latent variables. These help the encoder learn deeper data structures and more discriminative and generalized feature representations. The experiment results on the NTU-60 and NTU-120 datasets demonstrate that our proposed method can effectively improve the classification accuracy of the encoder in classification tasks and its generalization ability in the case of few labeled data. SkeletonMVAE exhibits stronger classification ability, while SkeletonMVQVAE exhibits stronger generalization in situations with fewer labeled data.

## 1 Introduction

Action recognition has consistently remained an active topic of research within the realm of computer vision. Compared with other data formats such as RGB (Simonyan and Zisserman, 2014; Feichtenhofer et al., 2019; Buch et al., 2017; Varol et al., 2017) and depth information (Cao et al., 2017; Fang et al., 2017; Xu et al., 2020; Chen et al., 2021), skeleton data (Duan et al., 2022; Thoker et al., 2021) eliminate the interference of redundant information such as background and lighting. It has the advantages of high order, lightweight, and high robustness. With the deepening of pose estimation (Cao et al., 2017; Lu et al., 2023) research, the extraction of human skeleton data has become more and more fast and accurate.

Nowadays, the mainstream human action recognition method is still fully supervised learning, which can be divided into recurrent neural network (RNN) (Du et al., 2015; Li et al., 2019), convolutional neural network (CNN) (Hou et al., 2016; Wang et al., 2016; Banerjee et al., 2020), graph convolutional neural network (GCN) (Yan et al., 2018; Liu Z. et al., 2020), and transformer (Wang et al., 2021; Zhang et al., 2021). These methods require a large amount of labeled data. However, it is a costly and time-demanding task to collect and label data. In addition, these methods may lead to overfitting during the learning process. To alleviate these problems, some works use self-supervised methods to learn unlabeled data. They mainly hope to learn a universal feature representation by solving pretext tasks, and then use it for downstream tasks such as contrastive learning (Dong et al., 2023; Lin et al., 2023). Contrastive learning allows the model to learn the feature invariance of the same skeleton sequences from different views by constructing positive and negative pairs through data augmentation. However, these methods of comparative learning pay more attention to global features, ignore the context relationship between frames, and depend on the number of comparison pairs.

Recently, action recognition introduced a new self-supervised method, mask encoding, and proved its effectiveness. In this method, a part of the data is masked, and the model infers the semantic information of the masked part through the context of the visible part, which can effectively capture the contextual relationship by analyzing the global and local information in the data, thereby enhancing the model's ability to capture intricate patterns and relationships within the data. MAE (He et al., 2022) has achieved success in the field of image. It can still effectively restore the original data by masking the image content with high probability. This excellent performance has attracted extensive research in different fields, and this concept has been applied to 3D human skeleton action recognition task. SkeletonMAE (Wu et al., 2023) based on human 3D skeleton sequence follows the idea of MAE. It randomly masks some frames and skeleton joints, uses the encoder—decoder structure to learn the relationship between unmasked skeleton joints, reconstructs the masked skeleton joints, and then uses the pre-trained encoder for human skeleton-based action recognition. However, the encoders of these methods can only encode limited features, and the extracted data information is not sufficient. To enhance the feature extraction ability and generalization ability of the encoder, we propose SkeletonMVAE, which inserts the potential space of the variational autoencoder (VAE) (Kingma and Welling, 2013) behind the encoder of SkeletonMAE. The latent variables of the variational autoencoder (VAE) are expressed in the form of distribution, allowing the encoder to learn deeper data structures and data distributions. By constraining the latent variables to a normal distribution close to the standard, the encoder can encode more discriminative feature representations, which is more conducive to the classification task. Furthermore, we propose SkeletonMVQVAE, which replaces the latent space with the latent space of the vector quantization variational autoencoder (VQVAE) (Van Den Oord et al., 2017). In the quantization process, the change of smaller action will also lead to a sharp change in the latent vector, so the latent vectors of the same category are forced to be expressed in a more compact and distinguishable form, which is beneficial to improve the accuracy of the encoder for classification tasks. The potential space of VAE and VQVAE will enable the encoder to capture the inherent uncertainty and variability in the data, so as to obtain a more robust, more expressive, and more generalized feature representation.

Specifically, in the pre-training stage, the input skeleton sequences are randomly masked in temporal and spacial dimensions, and then, the unmasked data are input into the network for the reconstruction of the masked part. Finally, the decoder is removed in the fine-tuning stage, and a simple output layer is added after the encoder to predict the skeleton data. In the experiment stage, we discuss the effects of masking rate, latent variable dimension, decoder dimension, and decoder depth on the recognition task and found the best combination. Experiment results show that our method is generalized and robust and effectively improves the accuracy of classification in downstream classification tasks.

In general, we have made the following contributions:

1) To improve the feature extraction ability of the encoder after the masked reconstruction task, we propose SkeletonMVAE and SkeletonMVQVAE, which insert the potential space of the variational autoencoder (VAE) and the vector quantization variational autoencoder (VQVAE) into SkeletonMAE, respectively. We discuss the differences between them.
2) We compare several mainstream models on the dataset NTU-60 and NTU-120. Experiments show that our models can effectively improve the accuracy of downstream classification tasks. SkeletonMVAE has obvious advantages.
3) We prove that our models still have good robustness and generalization ability under extremely few label data. SkeletonMVQVAE has more advantages in the case of fewer data labels.

## 2 Related work

### 2.1 Contrastive learning

In the self-supervised learning, most methods use contrastive learning (Zhang et al., 2022; Chen et al., 2022) that aims to enable the model to differentiate between various inputs in the feature space, distinguishing between similarities and dissimilarities. Research in this area typically involves creating positive and negative pairs through data augmentation, extracting representations via an encoder, and computing the similarity between two samples. Positive samples exhibit high similarity, while negative samples demonstrate low similarity. Previous comparative learning used normal enhancement to construct similar positive sample pairs. AimCLR (Guo et al., 2022) employs extreme data augmentation to obtain more diverse positive samples. CrosSCLR (Li et al., 2021) dugs positive sample pairs from similar negative samples, uses multi-view mining positive samples to learn cross-view consistency, and extracts more comprehensive cross-view features. SkeAttnCLR (Hua et al., 2023) focuses on the fact that human actions are often related to local body parts. Therefore, local salient features and non-salient features were proposed, and a large number of contrast pairs were generated to guide the model to learn the action representation of the human skeleton. The above contrastive learning usually has problems such as the need for a large number of comparison pairs and the lack of correlation between frames.

### 2.2 Masked encoding

Other self-supervised works such as BERT (Devlin et al., 2018), MAE (He et al., 2022), and SkeletonMAE (Wu et al., 2023) leverage masked reconstruction as a pretext task, which well enhance the learning of contextual relationships in data time and space. In natural language processing, the famous model BERT (Devlin et al., 2018) masks tokens representing sequential data and then predicted the masked tokens. It calculates the loss between the predicts results and the original data to capture the features of language sequences. Following the idea of BERT, in the field of image processing, MAE (He et al., 2022) adopts an asymmetric encoder—decoder structure to mask image patches and reconstructed them at the pixel level. Inspired by MAE, VideoMAE (Tong et al., 2022) applies masking encoding to the field of RGB video. Because of the redundancy of time, it can also bring good performance with a very high masking ratio. MAR (Qing et al., 2023) proposes “cell running masking” on the basis of VideoMAE to encourage the leakage of spatio-temporal information, hoping to use the redundancy of spatio-temporal to provide a detailed context for the encoder to reconstruct the missing patch. In the field of skeleton action recognition, SkeletonMAE (Wu et al., 2023) masks joints at the frame and joint levels, only encodes the unmasked joints and predicts the masked ones. This integration of masked reconstruction with self-supervised learning has shown promising results in various classification tasks and its potential to improve feature representation and classification performance.

### 2.3 The feature extraction of VAE and VQVAE

VAE and VQVAE have always been regarded as excellent generative models. Cheng et al. (2023) used VQVAE to generate coherent and structured fire scenarios, and the generated data were used for training and predicting wildfires. Zhu et al. (2023) proposed DSCVAE to generate consistent and realistic samples for predicting drop coalescence based on process parameters, improving prediction accuracy. With the development of deep learning, VAE and VQVAE have been used for feature extraction in many fields. Yue et al. (2023) uses the variational autoencoder to extract the feature invariance of EEG signals and then classifies them through a one-dimensional convolutional network. To extract the semantic features between words, Xu et al. (2023) use vaE to reconstruct the feature space so that it conforms to the normal distribution. This method can effectively extract text features for text classification. In the field of speech emotion recognition, TACN (Liu J. et al., 2020) proposes to use VQVAE to model speech signals and learn the intrinsic expression of datasets. Hsu et al. (2022) used VQVAE as the feature extraction module of the pre-training model to extract the spectral features of prosodic phrases. In the field of anomaly detection, LSGS (Wang et al., 2023) uses VQVAE to extract image features and locates anomalies by reconstructing a more accurate image. They introduced VAE and VQVAE as feature extractors to improve the performance of the model. Therefore, in the field of human skeleton-based action recognition, we introduce VAE and VQVAE into the self-supervised method of masking reconstruction. We hope to recover the masked data through its good generation ability to improve the feature extraction ability of the original encoder.

## 3 Methods

In this section, based on SkeletonMAE, we propose to improve the potential space of the masked reconstruction model. We explore two potential spatial patterns: one is the continuous potential space of VAE, and the other is the discrete potential space of VQVAE. We first review the characteristics of the two potential spaces and then introduce the network structure in detail.

### 3.1 The potential space of VAE and loss function

Given the skeleton joint dataset $X={\left\{x_{i}\right\}}_{i=1}^{N}$, which contains *N* samples. We make *z* obey the standard normal distribution, and the probability distribution of the reconstructed sample *x* of the decoder is $P\left(x\right)=\underset{z}{\int }P\left(z\right)P\left(x|z\right)dz$, where *P*(*z*) is the probability of sampling the encoded *z* from the standard normal distribution, and *P*(*x*|*z*) is the probability of the output sample *x* of the decoder when the encoded *z* is input. By maximizing $L=\underset{x}{\sum }logP\left(x\right)$, the reconstructed data are similar to the original data. However, not all *z* is meaningful, so *p*(*z*|*x*) is introduced to obtain the *z* corresponding to the input *x*. The posterior distribution *p*(*z*|*x*) is difficult to obtain, so we can use the encoder to fit the distribution *q*(*z*|*x*) of any *x*, then


(1) $$\begin{array}{l} logP\left(x\right)=\underset{z}{\int }q\left(z|x\right)logP\left(x\right)dz\\ \;=\underset{z}{\int }q\left(z|x\right)log\left(\frac{P\left(z,x\right)}{q\left(z|x\right)}\right)dz+KL\left(q\left(z|x\right)||P\left(z|x\right)\right)\\ \;\geq \underset{z}{\int }q\left(z|x\right)log\left(\frac{P\left(x|z\right)P\left(z\right)}{q\left(z|x\right)}\right)dz \end{array}$$

The right half of the above equation is the Evidence Lower Bound (ELBO). We express it as *L*_*b*_ and hope that it is as large as possible.


(2) $$\begin{array}{l} L_{b}=\underset{z}{\int }q\left(z|x\right)log\left(\frac{P\left(x|z\right)P\left(z\right)}{q\left(z|x\right)}\right)dz\\ \;=\underset{z}{\int }q\left(z|x\right)log\left(\frac{P\left(z\right)}{q\left(z|x\right)}\right)dz+\underset{z}{\int }q\left(z|x\right)log\left(P\left(x|z\right)\right)dz\\ \;=-KL\left(q\left(z|x\right)||P\left(z\right)\right)+\underset{z}{\int }q\left(z|x\right)log\left(P\left(x|z\right)\right)dz \end{array}$$

As *L*_*b*_ increases, *KL*(*q*(*z*|*x*)||*P*(*z*)) decreases, and $\underset{z}{\int }q\left(z|x\right)log\left(P\left(x|z\right)\right)dz$ increases. Since *P*(*z*) obeys the standard Gaussian distribution and *q*(*z*|*x*) obeys the Gaussian distribution, our SkeletonMVAE reconstruction loss can be written as


(3) $$\begin{array}{l} L_{mvae}=\beta *\frac{1}{N}\overset{N}{\underset{i=1}{\sum }}\frac{1}{2}\left(e^{\sigma_{i}}-\left(1+\sigma_{i}\right)+{\left(\mu_{i}\right)}^{2}\right)\\ \;+\frac{1}{N}\overset{N}{\underset{i=1}{\sum }}\left(||x_{i}-\hat{x_{i}}|{|}^{2}\right) \end{array}$$

where σ_*i*_ represents the variance of the i-th sample in the latent space, μ_*i*_ represents the mean in the latent space, and $\hat{x_{i}}$ is the reconstructed sample. Adjusting the value of β can affect the model's emphasis on reconstruction loss and KL divergence loss during training. We hope that the model is more inclined to focus on retaining the details and structural information of the data and pay more attention to retaining the specific characteristics of the input data when generating the data, so the value of β is set to 0.005. We add the first part of the loss function as a regularization term. When *z* is known to obey the standard normal distribution, the first part constrains *p*(*z*|*x*), that is, the latent variable is close to the standard normal distribution, which helps the encoder to learn a more compact and discriminative data representation. Moreover, the latent variable in the form of distribution rather than the single value in SkeletonMAE can enhance the robustness of the model to noise and abnormal data and has better generalization, which can adapt to datasets with different distributions. The second part aims to minimizing the disparity between the reconstructed data and the original data.

### 3.2 The potential space of VQVAE and loss function

Unlike the usual MAE, VQVAE do not directly use *z* as the input of the decoder but map it to a discrete vector *z*_*q*_ according to a set of codebooks. Through vector quantization technology, the continuous feature space is mapped to the discrete potential space, which helps to learn more meaningful feature representation and improve the model's ability to represent data. Because codebook is discrete, even if the input data *x* change slightly, the quantized latent variable *z*_*q*_ will change greatly (jump to another discrete vector). It forces the encoder to extract key information from the input data *x* for meaningful mapping in the discrete space. This mandatory information compression mechanism encourages encoders to learn more meaningful latent variable representations. In addition, due to its discreteness, redundant information is removed, and data of the same category will have a more compact representation, and the anti-interference ability is also enhanced.

Specifically, for skeleton sequence data, the encoder outputs a continuous vector *z*∈ℝ^*N*×*D*×*T*′ × *V*′^. The network learns a codebook *E* = *e*_1_, *e*_2_, *e*_3_...*e*_*K*_ (*E*∈ℝ^*D*×*K*^), *e* is the D-dimensional vector in the codebook, and *K* is the size of the codebook. VQVAE completes the mapping between the continuous vector *z* and the codebook *E* through the nearest neighbor search.


(4) $$\begin{array}{l} k=\underset{j}{\arg\min}||z_{i}-e_{j}|{|}_{2} \end{array}$$

where *j* is the index of the codebook vector closest to *z*_*i*_.


(5) $$\begin{array}{l} z_{i}=e_{k} \end{array}$$

The continuous vector *z* is mapped to the discrete vector $z_{q}\in {\mathbb{R}}^{N\times D\times T^{\prime }\times V^{\prime }}$.

The reconstruction loss is defined as


(6) $$L_{mvqvae}=log\;p\left(x|z_{q}\right)+{\left\|sg[z]-z_{q}\right\|}_{2}^{2}+\beta {\left\|z-sg\left[z_{q}\right]\right\|}_{2}^{2}$$

Among them, the first part is the reconstruction loss, which optimizes the encoder and decoder by reducing the error of the original sequence and the reconstructed sequence. The second part faces challenges due to the argmin operation on the feature vector during mapping, preventing gradient calculation. To train the latent space codebook, the L2 error between the encoder's output *z* and the latent space *e* is computed, with sg representing the stop gradient operation. In the third part, the L2 error between the encoder's output *z* and the corresponding potential space *e* is also calculated, but sg is applied to *e* to ensure the encoder's output aligns with the embedding space and avoids drastic changes (switching from one embedding vector to another). The β is the weight coefficient, and we set it to 0.25.

### 3.3 Model architecture

**Figure 1:**
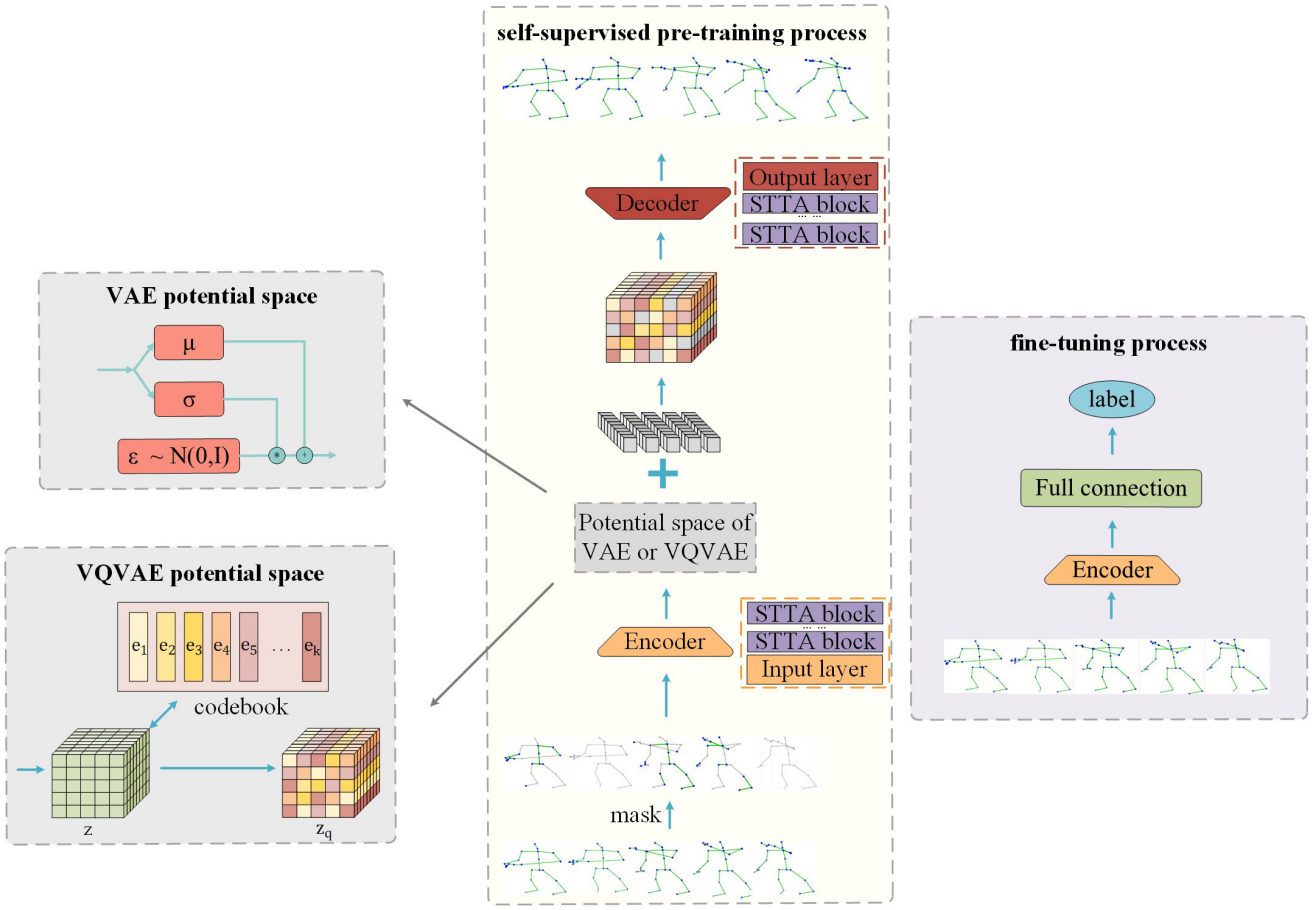
Masked reconstruction model structure.

We propose to insert the potential space of VAE and VQVAE into SkeletonMAE to improve the feature extraction ability of the encoder. The model structure is shown in Figure 1. The same thing of the SkeletonMVAE and SkeletonMVQVAE is that both have encoder and decoder. The encoder is employed to extract the feature representation of the unmasked data, while the decoder reconstructs the masked data based on the latent variables obtained during encoding. The difference is that SkeletonMVAE adds the potential spatial structure of VAE after the encoder, while SkeletonMVQVAE adds the potential spatial structure of VQVAE. The potential spatial structure of VAE and VQVAE is shown in Figure 1.

#### 3.3.1 Spatial-temporal masking strategy

**Figure 2:**
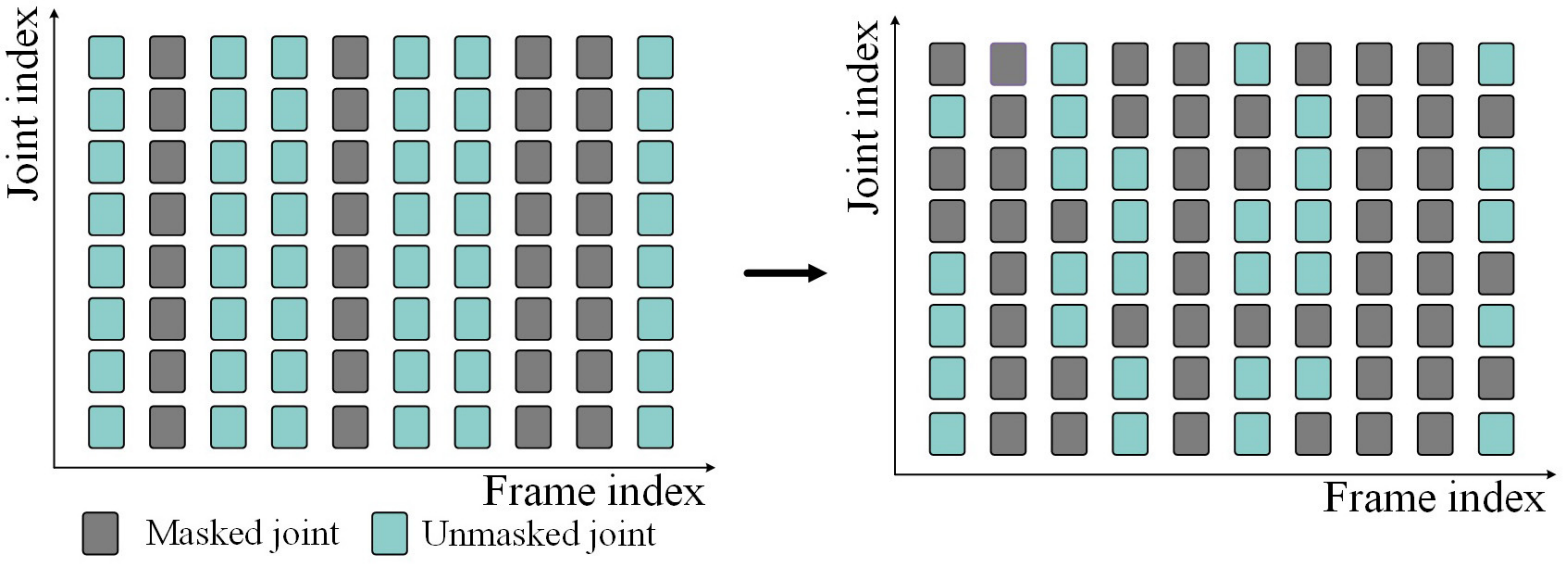
Spatial-temporal random masking process.

Because of the randomness of data loss, we perform random masking in both temporal and spatial dimensions when we mask skeleton data. Given the skeleton sequence *S*∈ℝ^*N*×*C*×*T*×*J*^. First, in the temporal dimensions, some frames are masked (i.e., deleted) according to the given frame masking rate *M*_*t*_, and the masked skeleton sequence becomes $S\in {\mathbb{R}}^{N\times C\times \left(1-M_{t}\right)\times T\times J}$. Then, in the spatial dimensions, the joints on all frames are randomly masked according to the given joint masking rate *M*_*j*_. Finally, the skeleton sequence input into the network is $S\in {\mathbb{R}}^{N\times C\times \left(1-M_{t}\right)\times T\times \left(1-M_{j}\right)\times J}$. The above masking process is shown in Figure 2. Gray represents the masked skeleton joints, and blue-green represents the unmasked skeleton joints.

#### 3.3.2 Encoder

As shown in Figure 1, our model applies multiple STTFormer blocks to capture the relationships between different keypoints in consecutive frames, which is used to encode and represent the unmasked parts of the skeleton sequence. To preserve the location information, we introduce location embedding after masking.

#### 3.3.3 SkeletonMVAE potential space

After the data pass through the encoder, the mean value μ and the standard deviation σ are output through the fully connected layer. The reparameterization technique is used to sample from the latent space to obtain the latent variables *z* = μ+σ*ε (ε~N(0,I)). This process is shown in Figure 1. As mentioned in Section 3.1, KL divergence, as a regularization term, limits the potential variables to approach the standard normal distribution. Regular potential space means that the Gaussian distribution parameters of the same category are basically the same after mapping to the potential space, and the adjacent points in the potential space are the same category after decoding. The encoder can learn the more compact and discriminative data representation, which is more conducive to the classification task.

#### 3.3.4 SkeletonMVQVAE potential space

Different from SkeletonMVAE, in SkeletonMVQVAE, the data pass through the latent space of VQVAE after the encoder. As described in Section B, in the latent space of VQVAE, the feature vector *z* is mapped to the discrete latent vector *z*_*q*_ according to the discrete codebook. This process is shown in Figure 1. The discrete potential space makes the encoder more able to extract representative features and enhance its robustness to noise.

#### 3.3.5 Decoder

As shown in Figure 1, our model decoder also consists of multiple STTFormer blocks. Feature vectors are sampled from the latent space formed by the encoder. We complement the learnable token for the missing skeleton sequence. The decoder decodes the masked token based on the sampled feature vectors and position information. The reconstruction goal is to be consistent with the original skeleton sequences.

### 3.4 Pre-train

The pre-training process of our model is shown in Figure 1. We randomly mask skeleton joints at both temporal and spatial dimensions and then add the positional embedding to the skeleton sequences. The unmasked skeleton data are fed into the encoder, mapped to the latent space. The sampled unmasked skeleton data, along with the mask token, are input into the decoder for reconstruction. When VAE potential space is used, we use the reconstruction loss of Equation 3 from Section A as the pre-training loss function. When VQVAE potential space is used, We use the reconstruction loss of Equation 6 from Section B as the pre-training loss function. The learning of the feature representation is continuously improved by minimizing the disparity between the original data and the reconstructed data. We save the model with the minimum verification loss as the best model.

### 3.5 Fine-tune

To evaluate the representation learning ability of our model, we utilize only the encoder part of the pre-trained model and add a fully connected layer for classification. We load the pre-trained parameter weights onto all training data and perform end-to-end fine-tuning for downstream recognition tasks. Throughout the fine-tuning process, the cross-entropy loss is utilized as the loss function and save the model with the maxmum verification accuracy as the best model.

## 4 Experiments

### 4.1 Datasets

#### 4.1.1 NTU-RGB+D 60

The NTU RGB+D 60 dataset (Shahroudy et al., 2016) contains 60 action classes with a total of 56,578 action sequences. Among them, there are 40 kinds of daily behavior actions, 9 kinds of health-related actions, and 11 kinds of mutual actions between two people. The dataset is partitioned into training and testing sets using two criteria. The first one is Cross-Subject, which divides the dataset into training set and test set based on different subject IDs. The training set and test set are completed by 20 different subjects, respectively, which are used to evaluate the performance of the model under different subjects of the same action. The second is Cross-View. The three cameras that capture the video are at the same height and different angles. The data of camera 1 are used in the test phase, and the data of camera 2 and camera 3 are used in the training phase, which can be used to evaluate whether the model can perform action recognition for skeletons at different angles.

#### 4.1.2 NTU-RGB+D 120

The NTU RGB + D 120 dataset adds 60 action categories based on NTU RGB+D 60 and adds 32 settings. Each setting uses different camera heights and different distances from the subject. The dataset is also divided using two criteria. The rule of Cross-Subject is consistent with NTU RGB+D 60. According to the ID of the subject, 53 people are divided into the training set and the other 53 people are divided into the test set. The model considers both different subjects and different settings during the training and testing process, which is used to evaluate the generalization ability of the model in the real world. In addition, it also adopts another partitioning strategy: Cross-Setup, which divides the training set and the test set according to the IDs of 32 settings. The even ID is classified as the training set, and the odd ID is classified as the test set, which is used to evaluate the adaptability of the model under different perspectives on the setting of the same subject.

### 4.2 Experiment settings

Our experiments are implemented under the framework of Pytorch (Paszke et al., 2019), using a computing node on the supercomputing platform and four HYGON DCUs under one computing node. Both the pre-training model and fine-tuning model utilize the Adam optimizer. When VAE potential space is used, the base learning rate is set to 0.001. When VQVAE potential space is used, the base learning rate is set to 0.01. We set the weight decay to 0.0001. The pre-training epoch number is 200, and the fine-tuning epoch number is 200. Batch size is set to 64. We employ a step-wise learning rate strategy, adjusting the learning rate to one-tenth at epochs 60, 90, and 110.

### 4.3 Comparison with existing mainstream methods

The comparison of our SkeletonMVAE, SkeletonMVQVAE, and other mainstream models on the NTU-60 and NTU-120 datasets is shown in Table 1. On the NTU-60 dataset, our SkeletonMVAE achieved a 1.8% higher accuracy than SkeletonMAE under the X-sub protocol and a 0.2% higher accuracy under the X-view protocol, and our SkeletonMVQVAE achieved a 1.4% higher accuracy than SkeletonMAE under the X-sub protocol and a 0.4% higher accuracy under the X-view protocol. On the NTU-120 dataset, our SkeletonMVAE achieved a 3.8% higher accuracy than SkeletonMAE under the X-sub protocol and a 4.4% higher accuracy under the X-set protocol, and our SkeletonMVQVAE achieved a 3.2% higher accuracy than SkeletonMAE under the X-sub protocol and a 2.3% higher accuracy under the X-set protocol. We can see that our SkeletonMVAE fine-tuning results is not only outperform other classical methods on small datasets but also have the potential to perform even better on larger datasets. The accuracy of our SkeletonMVQVAE on NTU-60 and NTU-120 datasets is also improved to varying degrees compared to SkeletonMAE. In this way, in terms of improving accuracy, SkeletonMVAE is slightly better, and the potential space of VAE-style regularization is more conducive to the realization of classification tasks. Experimental results show the effectiveness of our proposed method.

**Table 1:** Fine-tuned results on NTU-60 and NTU-120 datasets.

		**NTU-60 (%)**		**NTU-120 (%)**	
**Method**	**Backbone**	**X-sub**	**X-view**	**X-sub**	**X-set**
SkeletonCLR (Hua et al., 2023)	ST-GCN	82.2	88.9	73.6	75.3
CPM (Zhang et al., 2022)	ST-GCN	84.8	91.1	78.4	78.9
CrosSCLR (Li et al., 2021)	ST-GCN	86.2	92.5	80.5	80.4
AimCLR (Guo et al., 2022)	ST-GCN	86.9	92.8	80.1	80.9
Hi-TRS (Chen et al., 2022)	Transformer	86.0	93.0	80.6	81.6
AimCLR (Guo et al., 2022)	STTFormer	83.9	90.4	74.6	77.2
CrosSCLR (Li et al., 2021)	STTFormer	84.6	90.5	75.0	77.9
SkeletonMAE (Wu et al., 2023)	STTFormer	86.6	92.9	76.8	79.1
SkeletonMVAE	STTFormer	**88.4**	93.1	**80.6**	**83.5**
SkeletonMVQVAE	STTFormer	88.0	**93.3**	80.0	81.4

The bold values represent optimal values.

### 4.4 Semi-supervised results

We randomly sample 5% and 10% of data from the training set for semi-supervised fine-tuning. The sampling rule is to sample the same proportion of data within each class. The semi-supervised results are presented in Table 2. The results show that the performance of our proposed method is significantly better than the compared methods. We compare our SkeletonMVAE and SkeletonMVQVAE with the SkeletonMAE on NTU-60 and NTU-120 datasets. SkeletonMVAE has different degrees of improvement (0.5% to 3.5%) than SkeletonMAE. SkeletonMVQVAE also has different degrees of improvement (0.4% to 5.3%) than SkeletonMAE. It can be seen that when the sampling ratio is 5%, SkeletonMVQVAE shows a greater advantage. When the sampling ratio is 10%, SkeletonMVAE and SkeletonMVQVAE perform basically the same. It can be concluded that the potential space of SkeletonMVQVAE discretization is more conducive to generalization in the case of less labeled data. The experimental results indicate that our SkeletonMVAE and SkeletonMVQVAE still exhibit generalization ability with a small amount of labeled data and the generalization is improved compared to SkeletonMAE.

**Table 2:** Fine-tuned result comparison on the NTU-60 and NTU-120 datasets with fewer labeled data.

	**NTU-60 (%)**				**NTU-120 (%)**			
**Method**	**X-sub**		**X-view**		**X-sub**		**X-set**	
	**5%**	**10%**	**5%**	**10%**	**5%**	**10%**	**5%**	**10%**
Hi-TRS (Chen et al., 2022)	63.3	70.7	68.3	74.8	–	–	–	–
CrosSCLR (Li et al., 2021)	63.5	71.0	66.9	75.1	50.2	58.5	50.4	60.6
AimCLR (Guo et al., 2022)	63.9	70.2	67.5	76.2	49.0	58.6	51.8	60.5
SkeletonMAE (Wu et al., 2023)	64.4	73.0	68.8	76.9	50.4	61.8	52.0	62.5
CPM (Zhang et al., 2022)	–	73.0	–	77.1	–	–	–	–
SkeletonMVAE	65.1	**73.7**	67.1	**78.1**	53.9	**62.7**	53.0	**64.6**
SkeletonMVQVAE	**66.2**	73.5	**69.6**	77.9	**55.7**	62.4	**56.3**	64.3

The bold values represent optimal values.

### 4.5 Ablation study

All the experiments in this section are carried out on the NTU-60 dataset, and more detail of the model we proposed is analyzed.

#### 4.5.1 Frame and joint masking ratio

According to practical experience, the missing information in actual data is typically random. Therefore, we adopted a random method to mask the joints in both temporal and spatial dimensions. In temporal dimension, frames are masked with probabilities of 0.4, 0.5, and 0.6, while in spatial dimension, joints are masked with probabilities of 0.4, 0.6, and 0.8. As shown in Table 3, under the X-sub partition standard of NTU-60, when the frame mask rate is 0.4 and the joint mask rate is 0.4, SkeletonMVAE and SkeletonMVQVAE have the best performance.

**Table 3:** Ablation study on frame and joint masking ratio.

**Frame masking ratio**	**Joint masking ratio**	**SkeletonMVAE (%)**	**SkeletonMVQVAE (%)**
	0.4	88.4	88.0
0.4	0.6	87.0	86.2
	0.8	87.8	86.8
	0.4	87.5	86.5
0.5	0.6	87.5	87.8
	0.8	87.4	87.7
	0.4	87.4	86.1
0.6	0.6	87.5	87.3
	0.8	87.4	87.1

#### 4.5.2 Latent variable dimension

The dimension of the latent variable in the VAE determines the number of features that the model can learn and represent, which influence model's capacity to learn and represent features and the quality of the generated data. We conduct experiments with different latent variable dimensions, and the results are presented in Table 4. It shows that the model performs best when the latent variable dimension is 25. The lower latent variable dimension can lead to information loss, while the higher latent variable dimension can increase the model's complexity, requiring more training data and time to achieve good performance. Considering the model's performance and available resources, we chose the latent variable dimension of 25.

**Table 4:** Ablation study on SkeletonMVAE latent variable dimension.

**Latent variable dimension**	**SkeletonMVAE (%)**
15	87.7
25	88.4
35	87.5
45	87.6
55	86.9
65	87.6

We explore the impact of the codebook size of the latent space (i.e., the compactness of the latent vector) on the classification task. The influence of codebook size K on classification task is shown in Table 5. It is found that too large or too small K is not conducive to classification, because too large K has no way to learn compact representation, and too small K will lose information. When K is 128, the classification accuracy is 88.0%, which is the best result. The different latent space makes SkeletonMVQVAE greatly reduce the number of parameters of the model compared with SkeletonMVAE.

**Table 5:** Ablation study on SkeletonMVQVAE latent variable dimension.

**Latent variable dimension**	**SkeletonMVQVAE (%)**
100	86.8
128	88.0
256	86.3
512	87.7

#### 4.5.3 Decoder embedding dimension

We perform ablation experiments on the decoder's embedding dimension, evaluating the model's performance across three different dimensions: 128, 256, and 512. The experimental results are shown in Table 6. The SkeletonMVAE and SkeletonMVQVAE achieve the best accuracy when the decoder embedding dimension is set to 256.

**Table 6:** Ablation study on decoder embedding dimension.

**Dimension**	**SkeletonMVAE (%)**	**SkeletonMVQVAE (%)**
128	86.1	86.7
256	88.4	87.7
512	86.6	86.4

#### 4.5.4 Decoder depth

We further conduct the ablation study on different decoder depths (i.e., the number of STTFormer blocks used and a full connection layer). The decoder depths are set to 5, 7, 9, and 11 layers. The results are shown in Table 7. Too deep or shallow depths both reduce the fine-tuning accuracy. Considering the fine-tuning accuracy and model parameters, we set the decoder depth of SkeletonMVAE to 9 layers, and the decoder depth of SkeletonMVQVAE to 7 layers.

**Table 7:** Ablation study on decoder depth.

**Decoder depth**	**SkeletonMVAE (%)**	**SkeletonMVQVAE (%)**
5	87.9	87.4
7	87.5	88.0
9	88.4	87.7
11	87.9	87.1

As shown in Table 8, compared with SkeletonMAE, the parameters of SkeletonMVAE increased by 14M, the FLOPs increased by 2.1G, and the training time increased by 3 h. The parameters of SkeletonMVQVAE decreased by 1M, the FLOPs decreased by 6.5G, and the training time decreased by 1 h. SkeletonMVQVAE achieves the best performance when the decoder depth is 7, and the training efficiency is also improved compared with SkeletonMVAE.

**Table 8:** Number of model parameters, computational complexity, and pre-training time for SkeletonMVAE and SkeletonMVQVAE.

**Model**	**Parameter (M)**	**FlOPs (G)**	**Pre-training time (hours)**
SkeletonMAE	11	42.8	39
SkeletonMVAE	25	44.9	42
SkeletonMVQVAE	10	36.3	38

We summarize the Skeletonmvae network structure as shown in Table 9. The dim(input) is the output dim of block8 × (1−*M*_*t*_) × *T*×(1−*M*_*j*_) × *J*. The dim(output) is *C*×(1−*M*_*t*_) × *T*×(1−*M*_*j*_) × *J*.

**Table 9:** Structure of SkeletonMVAE.

	**Layer name**	**Input dim**	**Output dim**	**QKV dim**
Encoder	Input layer	3	64	
		Block1	64	64	16
		Block2	64	64	16
		Block3	64	128	32
		Block4	128	128	32
		Block5	128	256	64
		Block6	256	256	64
		Block7	256	256	64
		Block8	256	256	64
	vae(input)	Dim(input)	2*25	
	vae(output)	25	dim(output)	
Decoder	Block1	256	256	64
		Block2	256	256	64
		Block3	256	256	64
		Block4	256	128	64
		Block5	128	128	32
		Block6	128	64	32
		Block7	64	64	16
		Block8	64	64	16
		Output layer	64	3	

In addition, we also list the model structure of SkeletonMVQVAE, as shown in Table 10. SkeletonMVQVAE shows better results on fewer decoder layers. We believe that it is because too strong decoder is not conducive to the encoder to extract features. In this way, SkeletonMVQVAE further reduces the parameters of the model.

**Table 10:** Structure of SkeletonMVQVAE.

	**Layer name**	**Input dim**	**Output dim**	**QKV dim**
Encoder	Input layer	3	64	
		block1	64	64	16
		block2	64	64	16
		block3	64	128	32
		block4	128	128	32
		block5	128	256	64
		block6	256	256	64
		block7	256	256	64
		block8	256	256	64
Decoder	block1	256	256	64
		block2	256	256	64
		block3	256	128	64
		block4	128	128	32
		block5	128	64	32
		block6	64	64	16
		output layer	64	3	

## 5 Conclusion

The masked reconstruction model aims to improve the accuracy of the encoder in the downstream classification task by pre-training the reconstruction of the masked skeleton joints. The traditional masked reconstruction model uses the autoencoder structure but cannot learn richer potential information and data structure. To this end, we propose to improve the latent space based on the SkeletonMAE model and explore two different latent spaces. One is the VAE normal distribution regularization space, and the other is the VQVAE discrete latent space. We also performed pre-training of masked reconstruction and fine-tuning of downstream classification tasks on them and discussed the influence of two different latent spaces on downstream classification tasks, as well as the generalization ability of two different latent spaces. The experimental results show that the use of different latent spaces in pre-training can significantly improve the performance of downstream classification tasks in human skeleton-based action recognition. This shows that the choice of potential space plays a vital role in improving the overall effectiveness of the SkeletonMAE model. The shortcoming of this model is that the current focus is still on the field of action recognition, and the problem of cross-domain generalization needs further research to make it more conducive to practical application.

## Data Availability

The original contributions presented in the study are included in the article/supplementary material, further inquiries can be directed to the corresponding author.
