# Erratum: Latent space improved masked reconstruction model for human skeleton-based action recognition

**DOI:** 10.3389/fnbot.2025.1587250

**Published:** 2025-03-18

**Authors:** Frontiers Production Office

**Keywords:** human skeleton-based action recognition, variational autoencoder, vector quantized variational autoencoder, masked reconstruction model, self-supervised learning

Due to a production error, the date in the DOI for this published article was erroneously written as “2024” instead of the correct version “2025” in the citation section and PDF file header. The correct DOI for this published article appears below:

doi: 10.3389/fnbot.2025.1482281

The publisher apologizes for this mistake. The original version of this article has been updated.

